# Single-Step Microfluidic Anti-Factor Xa Assay: Development and Clinical Evaluation in Patients on Low-Molecular-Weight Heparin

**DOI:** 10.3390/s26216803

**Published:** 2026-10-28

**Authors:** Stacia Shi En Lim, Yuxi Wang, Jasmine Shu Hui Lim, Jeanie Jing Yi Yeo, Christina Lai Lin Sum, Mui Ka Ang, Bernard Pui Lam Leung, Bingwen Eugene Fan, Yi Fu

**Affiliations:** 1 Healthcare Engineering Centre, School of Engineering, Temasek Polytechnic, 21 Tampines Avenue 1, Singapore 529757, Singapore; 2 Department of Laboratory Medicine, Tan Tock Seng Hospital, 11 Jalan Tan Tock Seng, Singapore 308433, Singapore; 3 Cluster of Health and Social Sciences, Singapore Institute of Technology, 1 Punggol Coast Road, Singapore 828608, Singapore; 4 Department of Rheumatology, Allergy and Immunology, Tan Tock Seng Hospital, Singapore 308433, Singapore; 5 Department of Haematology, Tan Tock Seng Hospital, 11 Jalan Tan Tock Seng, Singapore 308433, Singapore; 6 Klaris Precision Health Clinic, Tan Tock Seng Hospital, 11 Jalan Tan Tock Seng, Singapore 308433, Singapore; 7 Lee Kong Chian School of Medicine, Nanyang Technological University, 11 Mandalay Road, Singapore 308232, Singapore; 8 Northern Clinical Diagnostics & Thrombovascular Research (NECTAR), Northern Health, 185 Cooper Street, Epping, Melbourne, VIC 3076, Australia; 9 Department of Medicine, University of Melbourne, Grattan Street, Parkville, VIC 3010, Australia

**Keywords:** microfluidics, anti-factor Xa assay, point-of-care testing, colorimetric sensing, anticoagulant monitoring

## Abstract

Direct oral anticoagulants and low-molecular-weight heparins (LMWHs) act principally through inhibition of activated factor X (FXa), yet no point-of-care assay for anti-FXa activity is in routine clinical use. This study reports a disposable microfluidic device that performs a single-step colorimetric anti-FXa assay on plasma. The chip was fabricated by laminating laser-cut poly(methyl methacrylate) sheets with adhesive tape; both assay reagents were pre-stored on-chip in dried form, and absorbance was measured at 405 nm. In 58 plasma samples from patients receiving LMWH therapy, the on-chip result correlated moderately with the automated laboratory assay. Benchmarking against the same reagents in a microwell plate showed that the device recovered 46% of the attainable agreement. The microfluidic assay identified samples at or above a therapeutic anti-Xa concentration with 94% specificity and a positive predictive value of 96%. The device is therefore suited to rapid screening prior to laboratory confirmation.

## 1. Introduction

Anticoagulants are pharmacological agents that are used clinically to disrupt the coagulation cascade, thereby preventing thrombus formation, propagation, and subsequent embolic events. These drugs are essential for the treatment of thromboembolic disorders, which constitute one of the leading causes of morbidity and mortality worldwide.

**Figure 1:**
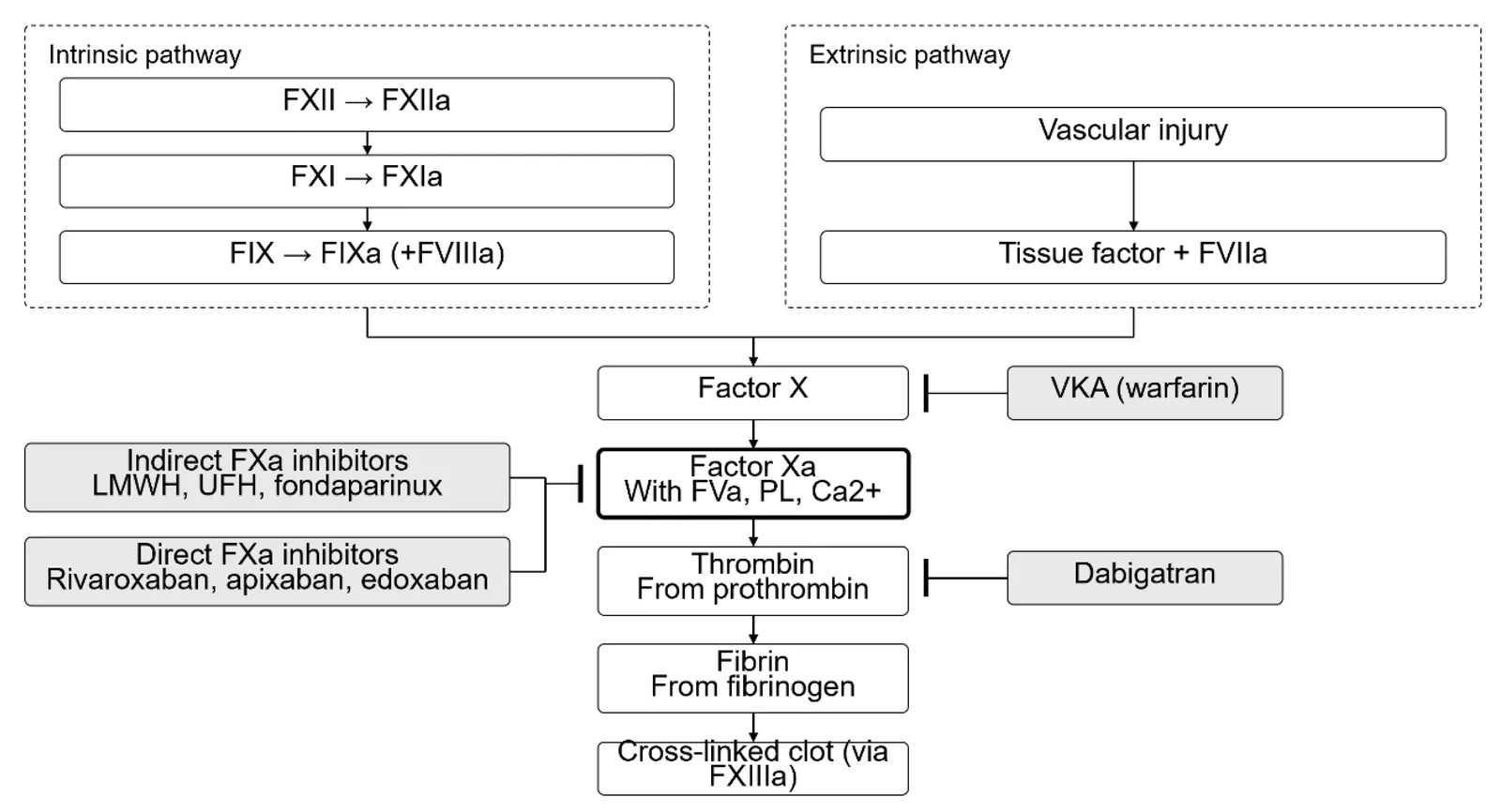
Coagulation cascade and sites of action of the main anticoagulant classes. Arrows denote activation steps and blunt-ended connectors denote inhibition; shaded boxes identify the anticoagulant classes, and dashed outlines group the intrinsic and extrinsic pathways.

Anticoagulant agents act by inhibiting specific pathways of the coagulation cascade. While anticoagulants like unfractionated heparin and warfarin remain relevant clinically, low-molecular-weight heparins (LMWHs) and direct oral anticoagulants (DOACs) now serve as the preferred frontline therapies across most clinical indications. These agents act preferentially on FXa rather than on thrombin (FIIa) and have been reported to provide an improved risk–benefit profile in comparison with conventional therapy (Figure 1) [1,2].

Owing to the narrow therapeutic window of LMWH and the adverse effects of bleeding or increased risk of thrombosis associated with inaccurate dosing, anticoagulant therapy requires careful monitoring. Conventional monitoring of warfarin and heparin is based on clot-based assays, namely prothrombin time (PT) and activated partial thromboplastin time (aPTT). These assays, however, are of limited sensitivity for the assessment of FXa-mediated anticoagulation in patients receiving DOACs or LMWHs [3]. The clinical value of routine anti-Xa monitoring remains a subject of debate. The American College of Chest Physicians (ACCP) 9th edition parenteral anticoagulant guideline states that routine monitoring is not generally necessary for LMWH but should be considered in obesity, renal insufficiency, and pregnancy, using peak anti-Xa activity measured four hours after dosing as the preferred test [4]. The principal application of rapid anti-Xa measurement is therefore considered to be urgent clinical decision-making rather than routine dose titration, since determination of whether a clinically relevant drug level is present may alter immediate patient management.

The plasma anti-FXa assay was developed as a functional test for the measurement of drug levels in patients receiving anti-Xa anticoagulants [5]. Point-of-care testing (POCT) devices are established for the measurement of PT and aPTT, but no equivalent commercial rapid point-of-care assay for anti-FXa activity has been established for routine clinical use. A recent systematic review of commercially available POCT systems for the rapid assessment of DOAC activity identified only clot-based and viscoelastic platforms, none of which measures anti-Xa activity directly [6]. An unmet clinical need therefore exists, particularly in view of the increasing use of the newer anticoagulant agents.

Microfluidic and lab-on-a-chip technologies provide a possible solution, since multiple laboratory functions can be integrated onto a miniaturized platform, and small sample volumes can be analyzed automatically at or near the patient’s bedside [7]. Realizing this potential for a multi-reagent enzymatic assay requires two capabilities in combination: a fabrication route that is low in cost and tolerant of rapid design iteration, and a method for storing the assay reagents on the device so that the analytical sequence proceeds without operator intervention. In the present study, a disposable single-use microfluidic device incorporating a colorimetric anti-FXa assay is described, in which laser-cut lamination is combined with the on-chip storage of both reagents in dried form. The validation data characterize the performance of the device in the measurement of LMWH activity in plasma samples and indicate its potential for POCT applications.

## 2. Materials and Methods

### 2.1. Liquid Anti-Xa Assay

**Figure 2:**
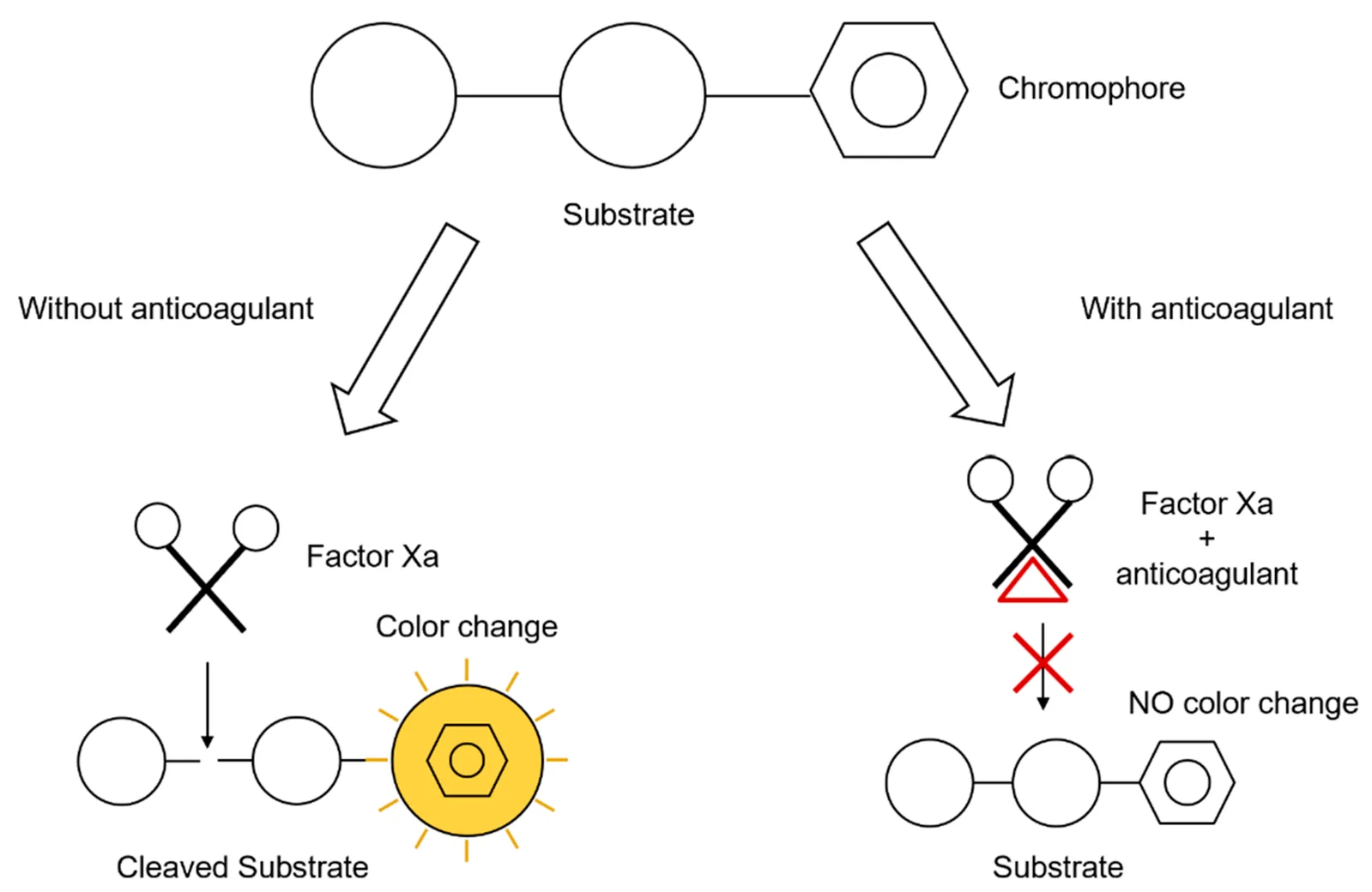
Principle of the chromogenic anti-Xa assay. In the absence of anticoagulant, FXa cleaves the chromogenic substrate and releases the chromophore, producing color at 405 nm; when FXa is inhibited, the substrate remains intact, and no color develops.

The activity of most DOACs and LMWHs is dependent on their capacity to inactivate FXa, a key enzyme of the blood coagulation pathway. A chromogenic assay was employed for the measurement of anti-Xa activity. In this assay, a chromophore-linked peptide substrate of FXa is used. Cleavage of the substrate releases a colored compound, whereas the reaction is inhibited in the presence of anticoagulant. The concentration of anticoagulant is therefore inversely correlated with the color intensity of the assay product (Figure 2).

The STA-Liquid Anti-Xa kit was obtained from Stago (Asnières-sur-Seine, France) and was used in accordance with the instructions of the manufacturer. For the manual assay, 10 µL of plasma sample or HEP calibrators diluted 1:4 with phosphate-buffered saline (PBS) were mixed sequentially with 50 µL each of FXa solution and chromogenic substrate in a standard microtiter plate. The mixture was subsequently incubated at 25 °C for 30 min for color development. Results were quantified by measurement of the optical absorbance of the reaction liquid at 405 nm using a microplate reader (TriStar^2^ LB 942, Berthold Technologies, Bad Wildbad, Germany).

Standard curves were constructed using STA Multi-HEP calibrators (Stago, Asnières-sur-Seine, France), which consist of artificial plasma spiked with defined concentrations of heparin. The anti-Xa activity of each calibrator is expressed in international units per milliliter (IU/mL). One international unit is defined as the quantity of heparin required to prevent clotting under standard assay conditions, with reference to the WHO International Standard. Calibrator activities ranged from 0 to 1.76 IU/mL.

### 2.2. Microfluidic Anti-Xa Assay

**Figure 3:**
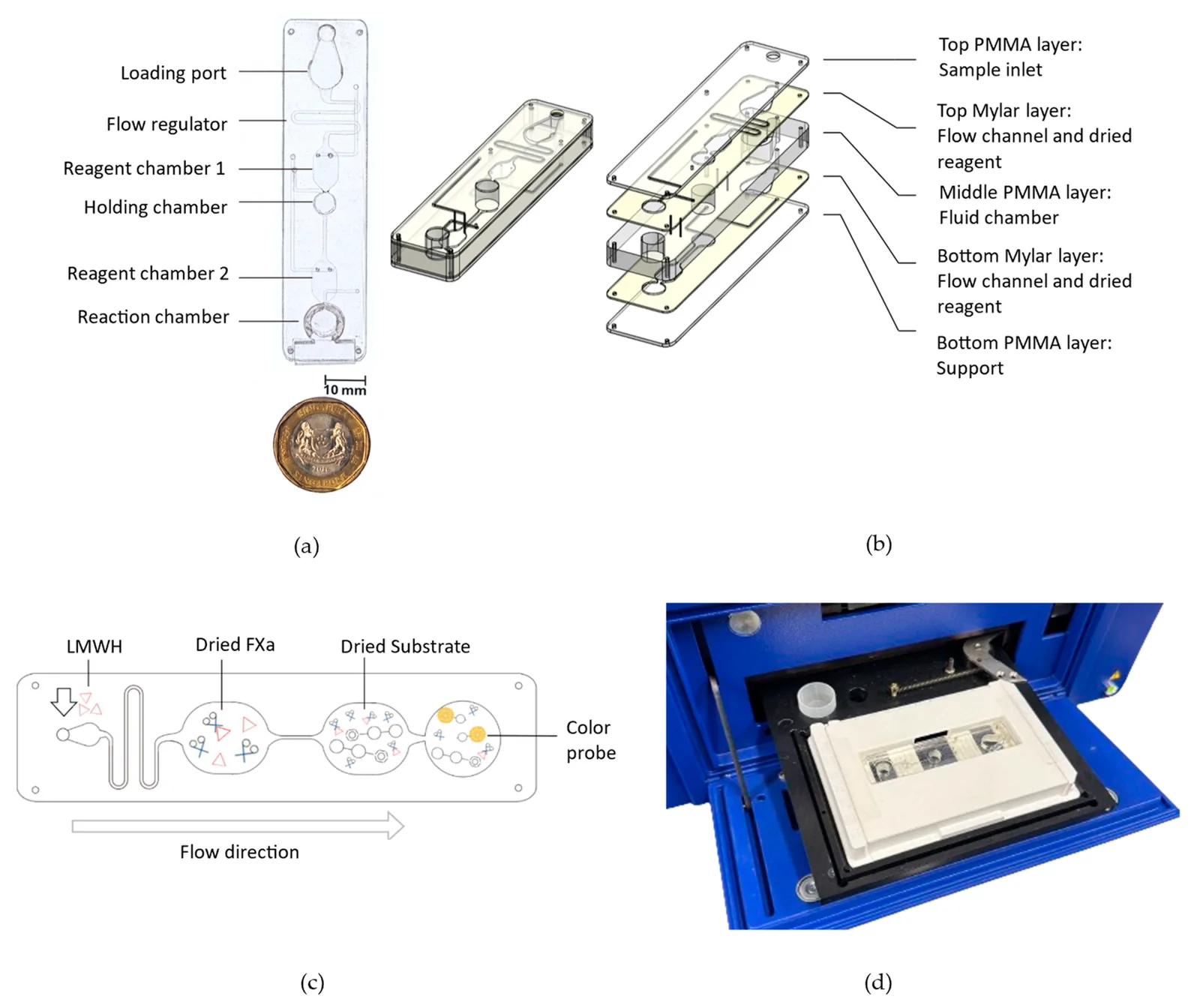
Microfluidic anti-Xa chip: (**a**) photograph of the assembled chip with the fluidic components labeled, shown alongside a one-dollar coin for scale (scale bar 10 mm); (**b**) three-dimensional rendering of the assembled device and exploded view of the five laminated layers, comprising the top PMMA layer carrying the sample inlet, the top Mylar layer defining the flow channel and holding one dried reagent, the middle PMMA layer forming the fluid chambers, the bottom Mylar layer defining the flow channel and holding the second dried reagent, and the bottom PMMA support; (**c**) schematic of the on-chip assay sequence, in which plasma containing LMWH enters at the loading port, reconstitutes the dried FXa in the first reagent chamber and then the dried chromogenic substrate in the second, color developing in the reaction chamber where absorbance is read, with the arrow indicating the direction of flow; (**d**) chip positioned in the customized jig within the microplate reader.

The microfluidic assay chip was constructed from three layers of poly(methyl methacrylate) (PMMA, 1 mm thick for the top and bottom layers and 6 mm for the middle layer) and two layers of 3M Mylar adhesive tape (250 µm thick). The assembled device carries a sample loading port, a central flow channel, a flow regulator, two reagent chambers, and a reaction chamber (Figure 3a). Flow structures were cut into the Mylar tape layers, whereas the inlet, outlet, and liquid chambers were formed in the PMMA layers, giving the five-layer stack shown in Figure 3b. Designs were prepared in CorelDRAW X7 and cut using an Epilog Laser Mini 24 engraver (40 W, 610 × 305 mm bed; Epilog Laser, Golden, CO, USA); this cut-and-laminate approach based on pressure-sensitive tape represents an established low-cost alternative to photolithographic fabrication [8]. The individual layers were aligned manually and laminated to form the chip. Prior to placement of the top and bottom covers, 75 µL aliquots of FXa reagent and chromogenic substrate were dispensed into the respective reagent chambers and dried under vacuum for 2 h. On-chip storage of reagents in dried form is a widely applied strategy for the elimination of sample-handling steps in point-of-care cartridges [9]. All chips used in the present study were fabricated manually. The reagents were dried from their supplied ready-to-use liquid formulations, without added lyoprotectants or stabilizers. Separate testing confirmed that drying and reconstitution do not compromise the activity of either reagent when assayed immediately after preparation (Supplementary Figure S1 and Table S1).

The sequence of events on the chip is shown schematically in Figure 3c. For each measurement, 200 µL of patient plasma, diluted 1:40 with PBS, was introduced through the loading port. This corresponds to the final plasma dilution of the microwell assay, in which 10 µL of a 1:4 pre-dilution was combined with 100 µL of reagents. As the on-chip reagents were dried and added with no volume, the sample was brought to the same final dilution in a single step. The chip was held upright so that the liquid flowed into the first reagent reservoir, where the dried FXa was reconstituted, and any anticoagulant present in the sample was able to inhibit the enzyme. Mixing was enhanced in the subsequent holding chamber. The liquid then advanced into the second reservoir, dissolving the dried chromogenic substrate, which the residual uninhibited FXa cleaves. Once the liquid had reached the reaction chamber, the chip was incubated at 25 °C for 30 min to allow the enzymatic reaction to proceed. Incubation at 25 °C was chosen because it corresponds to normal air-conditioned laboratory temperature, allowing the assay to be performed on the bench without an incubator or other heating element. The 30 min interval was adopted for consistency with the manual microwell and automated analyzer protocols, which follow the reagent manufacturer’s instructions, so that the three methods could be compared under equivalent reaction conditions. Following incubation, the chip was placed in a customized jig, and the absorbance of the assay liquid in the reaction chamber was measured at 405 nm using the TriStar^2^ LB 942 microplate reader (Figure 3d). The total time from sample loading to result was approximately 40 min.

### 2.3. Clinical Study Design

The clinical study was conducted at Tan Tock Seng Hospital; ethics approval was obtained from the National Healthcare Group Domain Specific Review Board (NHG DSRB Ref: 2023/00789). The requirement for informed consent was waived as the study used residual clinical specimens from which no patient-identifiable information was retrieved. Plasma samples were collected from patients on low-molecular-weight heparin therapy. Each sample was analyzed using the STA-Liquid Anti-Xa reagent on an automated coagulation analyzer (STA-R Max, Stago, Asnières-sur-Seine, France), by the manual microwell assay, and by the microfluidic chip assay. Agreement between methods was assessed by ordinary least-squares linear regression, with Pearson correlation coefficients and 95% confidence intervals from Fisher z-transformation; Spearman rank correlation, and Deming and Passing–Bablok regressions were calculated as checks [10,11]. For Bland-Altman analysis, on-chip absorbance was converted to concentration using a calibration against the analyzer fitted with leave-one-out cross-validation so that each predicted value came from a model excluding that sample. Confidence intervals for sensitivity, specificity, and predictive values were calculated by the Wilson score method, and for the area under the curve by bootstrap resampling with 4000 replicates. Classification performance was assessed by receiver operating characteristic (ROC) analysis, reporting the area under the curve (AUC) and the sensitivity, specificity, and predictive values at the Youden-optimal cut-off. Calibrator replicates were used to calculate precision and detection limits, and linearity was checked by the lack-of-fit F-test. On-chip absorbance in the patient samples was compared with that of the zero-heparin calibrator using Welch’s unequal-variance *t*-test. Analyses were performed in Microsoft Excel and Python 3 (NumPy, SciPy). Data figures were prepared with Matplotlib 3.10.

## 3. Results

### 3.1. Assay Response and Precision

**Figure 4:**
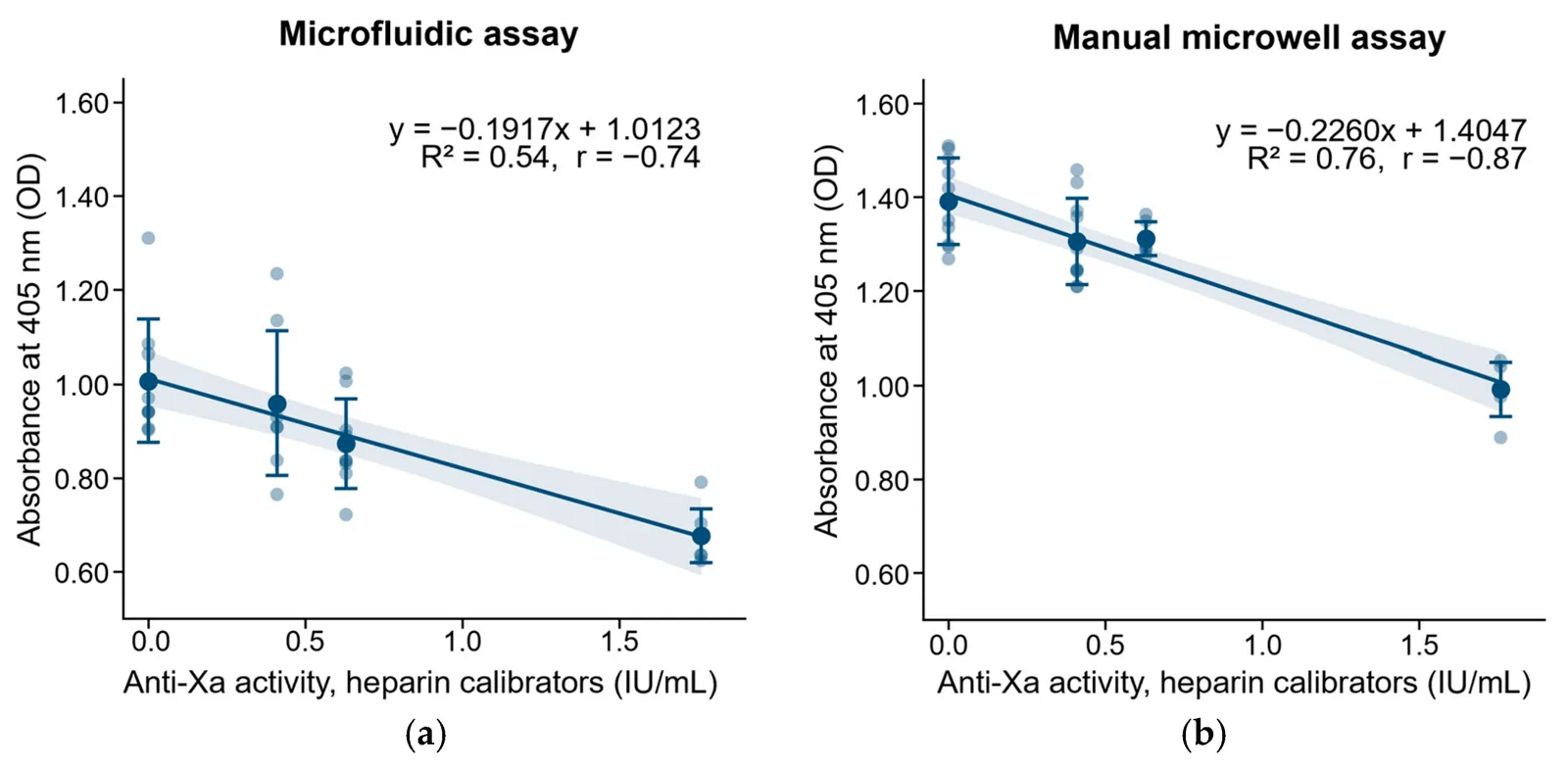
Anti-Xa standard curves obtained with STA Multi-HEP calibrators: (**a**) microfluidic assay; (**b**) manual microwell assay. Heparin activity is expressed in IU/mL and color intensity as optical density (OD) at 405 nm.

The assay was first characterized with heparin calibrators at four activities (0, 0.41, 0.63, and 1.76 IU/mL), each measured in seven to ten independent runs (Table S2). In both formats, absorbance decreased linearly as heparin activity increased, confirming that FXa was successfully inhibited within the chip (Figure 4). The response was shallower on-chip, and the measurements scattered more widely about the fitted line: the calibration accounted for 54% of the variation in absorbance on-chip, compared with 76% in the microwell plate. A lack-of-fit F-test gave no evidence of departure from linearity in either format (*p* = 0.74 on-chip and *p* = 0.26 in the plate), so a straight-line calibration is appropriate across this range. The two formats also differed in reproducibility: the coefficient of variation at individual calibrator concentrations was 8.5–16.0% on-chip compared with 2.7–7.1% in the microwell plate (Table S3). Detection and quantitation limits were estimated from the standard deviation of the zero-heparin calibrator divided by the slope of the calibration line, taken as 3.3 and 10 standard deviations, respectively. On this basis, the limit of detection was 2.25 IU/mL on-chip and 1.35 IU/mL in the microwell plate, with corresponding limits of quantitation of 6.81 and 4.09 IU/mL. These values exceed the highest calibrator used (1.76 IU/mL) and are therefore extrapolated estimates rather than experimentally validated analytical limits; they indicate the resolution implied by the observed blank variability and calibration slope and were not verified by measurement at those activities. Both values lie above the therapeutic range of 0.5 to 1.0 IU/mL, meaning that a single on-chip measurement cannot reliably separate a low heparin concentration from none. For this reason, the device was evaluated for its ability to sort samples into groups rather than to report a concentration.

### 3.2. Clinical Evaluation

**Figure 5:**
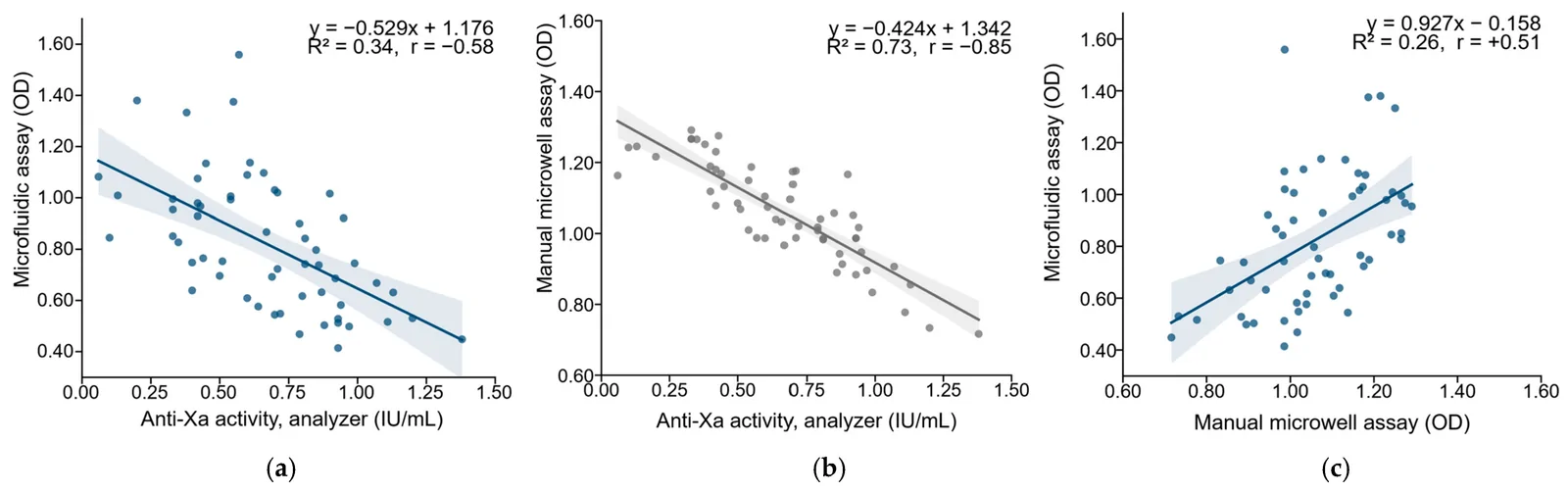
Clinical evaluation results: (**a**) correlation between microfluidic assay OD and anti-Xa activity measured on the automated analyzer; (**b**) correlation between manual microwell assay OD and the same analyzer result; (**c**) correlation between microfluidic and manual microwell assay OD. Shaded bands are 95% confidence intervals of the fitted regression line.

Fifty-eight samples were analyzed using the assay protocol in which the diluted plasma reconstituted the dried FXa before reaching the chromogenic substrate. Anti-Xa activity measured on the automated analyzer ranged from 0.06 to 1.38 IU/mL. On-chip absorbance decreased with increasing anti-Xa activity, accounting for 34% of the variance in the analyzer result (R^2^ = 0.34; r = −0.58, 95% CI −0.73 to −0.38; Figure 5a). The manual microwell assay, performed on the same samples with the same reagents, showed the same inverse relationship but tracked the analyzer more closely (R^2^ = 0.73; r = −0.85, 95% CI −0.91 to −0.77; Figure 5b). Figure 5c compares the two colorimetric formats directly, without reference to the analyzer; the two agreed with each other to R^2^ = 0.26 (r = 0.51, 95% CI 0.28 to 0.68), indicating that the scatter is not confined to either format alone.

Two statistical checks were applied to the correlation between the microfluidic assay and the analyzer shown in Figure 5a. A non-parametric Spearman’s rank correlation (rho = −0.62) showed that the association was not driven by a small number of extreme values. Error-in-variables regression, which allows for measurement error in the reference result as well as in the on-chip reading [10,11], returned slopes at least as steep as the ordinary least-squares fit, confirming that the strength of the relationship has not been overstated.

While the on-chip coefficient of determination (R^2^ = 0.34) is moderate, this value must be viewed in the context of what the assay chemistry itself allows. The manual microwell assay provides a direct benchmark: using the exact same reagents, analyte, and optical readout, it reached an R^2^ of 0.73 against the reference analyzer. This indicates that the microfluidic device recovers 46% of the attainable correlation. Two key points follow this comparison. First, the realistic performance ceiling for this assay is R^2^ = 0.73, not theoretical unity, and the device should be judged against this practical limit. Second, because the microwell format introduces significant variance on its own, much of the observed scatter clearly stems from the inherent chemistry and handling steps, such as sample dilution and incubation timing, rather than the microfluidic platform. Therefore, any future chip optimizations will only be able to address the device-specific subset of the overall variance.

To establish the influence of reagent sequence, a further 62 samples were analyzed with the order of the two dried reagents reversed so that the plasma reconstituted the chromogenic substrate before reaching the FXa. Agreement with the analyzer was substantially weaker (R^2^ = 0.17), and the absorbance range was narrower, 0.37–0.91 OD compared with 0.41–1.56 OD from the first protocol (Figure S2). A plausible explanation is that the first sequence permits the anticoagulant to inhibit FXa before substrate becomes available, whereas in the reversed sequence, substrate and inhibitor compete for the enzyme from the moment of reconstitution. The reversed sequence was not pursued further, and all subsequent analyses refer to the assay protocol described above.

### 3.3. Diagnostic Performance

Using the equation derived from the heparin standard curve, back-calculated anti-Xa activities for 55% of the patient samples fell outside the calibrator range, and the mean on-chip absorbance of the patient samples differed significantly from that of the zero-heparin calibrator (Welch’s unequal-variance *t*-test, *p* = 0.003). These discrepancies indicate a substantial matrix effect, wherein endogenous plasma constituents interfere with the optical readout independently of heparin concentration. For subsequent analyses, calibration was instead derived from the clinical samples themselves using leave-one-out cross-validation. This provides a concentration scale on which device performance can be compared directly with the reference laboratory assay.

**Figure 6:**
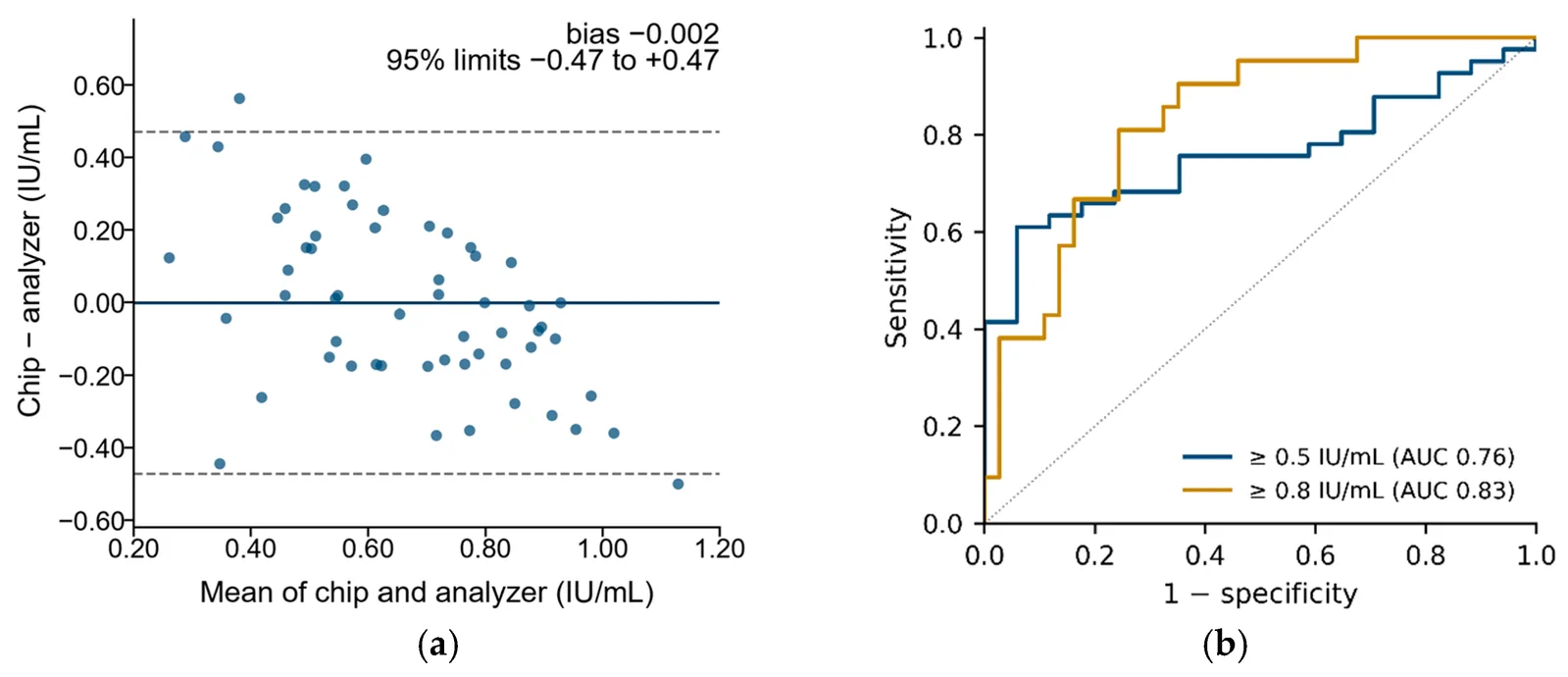
Agreement and classification performance of the microfluidic assay: (**a**) Bland–Altman plot after leave-one-out internal calibration, showing the mean difference (solid line) and the 95% limits of agreement (dashed lines); (**b**) receiver operating characteristic curves for the two reference thresholds.

Using this internal calibration, the device agreed closely with the analyzer on average, the mean difference across the samples being −0.002 IU/mL. Individual measurements were nevertheless widely dispersed, with 95% of results falling within ±0.47 IU/mL of the analyzer value (Figure 6a). The absence of a systematic offset indicates that the on-chip reaction itself is correctly calibrated, so the dispersion reflects random variation rather than bias. Its two sources are those identified in the previous section: the chromogenic chemistry and the device. Against a therapeutic range of approximately 0.5 to 1.0 IU/mL, dispersion of this magnitude precludes the reporting of a definitive concentration, and the assay was therefore evaluated for its ability to discriminate samples above and below clinically relevant anti-Xa thresholds. Converting the on-chip absorbance into concentration units was necessary only for the agreement analysis above. The classification results reported below are based on the absorbance readings themselves and involve no conversion, so they are unaffected by this step. Because the relationship used for the calculation was derived from the same 58 samples to which it was then applied, this analysis does not constitute independent quantitative validation of the device. The limits of agreement given here are the narrowest that would be expected, and a wider spread should be anticipated in samples not used to establish it.

Samples were categorized against two reference anti-Xa activity thresholds. The primary threshold of 0.5 IU/mL represents the lower limit of the peak target range for twice-daily therapeutic LMWH [12] and serves as the clinical decision point separating therapeutic from subtherapeutic anticoagulation. A secondary threshold of 0.8 IU/mL, which falls within the same target range, was used to determine whether discrimination improved at a higher cut-off. The area under the receiver operating characteristic curve was 0.76 (95% CI 0.64 to 0.87) at the lower threshold and 0.83 (95% CI 0.71 to 0.93) at the higher (Figure 6b), corresponding to acceptable and good discrimination, respectively, under conventional criteria. Classification performance therefore exceeded quantitative agreement.

**Figure 7:**
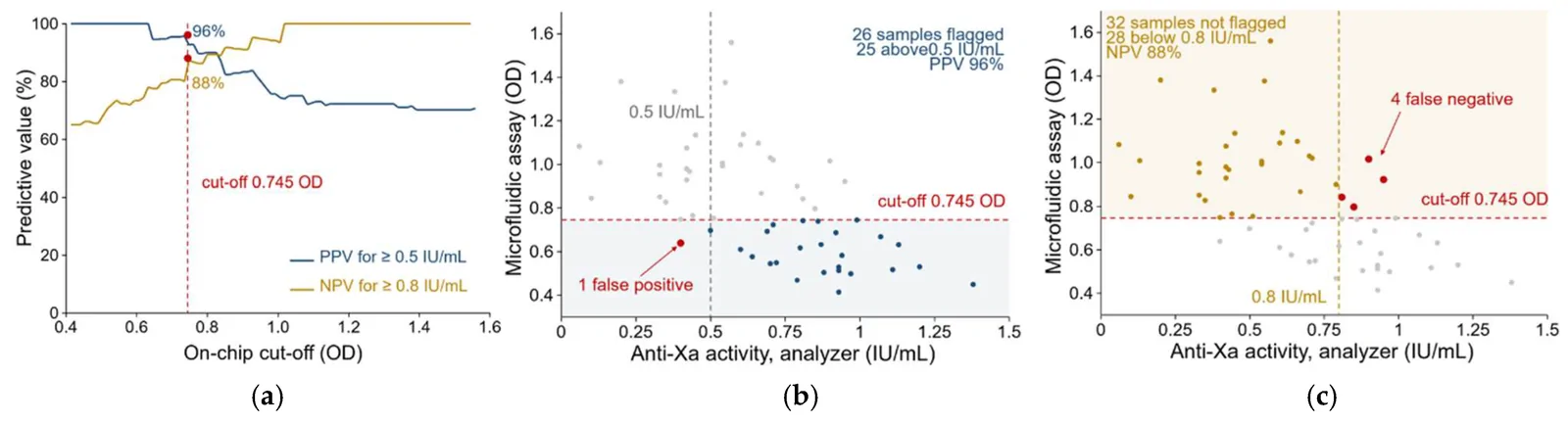
Diagnostic performance of microfluidic anti-Xa assay: (**a**) positive predictive value for anti-Xa activity of at least 0.5 IU/mL and negative predictive value for at least 0.8 IU/mL, across the range of possible cut-offs, with the chosen cut-off marked; (**b**) classification of the 58 samples at the on-chip cut-off of 0.745 OD against reference thresholds of 0.5 IU/mL, the shaded region contains samples flagged positive by the device; (**c**) classification against reference thresholds of 0.8 IU/mL, the shaded region contains samples flagged negative by the device. In (**a**), the blue curve shows the positive predictive value and the amber curve the negative predictive value, with the red dashed line marking the chosen cut-off; in (**b**,**c**), colored points are the samples contributing to the quoted predictive value, red points are misclassifications, and grey points fall outside the group concerned.

The same on-chip cut-off of 0.745 OD was optimal against both reference thresholds. Against the 0.5 IU/mL threshold, a result at or below the cut-off correctly identified 25 of the 26 samples flagged, corresponding to a positive predictive value of 96% (95% CI 81 to 99%) and a specificity of 94% (95% CI 73 to 99%); sensitivity was 61% (95% CI 46 to 74%), and the negative predictive value was 50% (95% CI 34 to 66%). Against the 0.8 IU/mL threshold, a result above the cut-off correctly excluded 28 of 32 samples, a negative predictive value of 88% (95% CI 72 to 95%), with a sensitivity of 81% (95% CI 60 to 92%) and a specificity of 76% (95% CI 60 to 87%) (Figure 7). As the cut-off was selected using these same 58 samples, these values are likely to be optimistic, and the confidence intervals are correspondingly wide.

A positive on-chip result is therefore consistent with the presence of a therapeutic concentration, whereas a negative result does not exclude anticoagulation: sensitivity was 61%, and only half of the samples not flagged were genuinely below 0.5 IU/mL. These values should be read alongside the moderate agreement on the concentration scale reported above (R^2^ = 0.34), which limits how strongly any screening claim can be stated. This asymmetry characterizes the present analytical performance of the device. Within a retrospective series of 58 samples and without clinical outcome data, these results establish analytical feasibility and support a preliminary screening role, with clinical utility to be confirmed prospectively.

## 4. Discussion

### 4.1. Origin of the Observed Variability

The moderate correlation with the reference laboratory analyzer arises from two separable sources. As established above, benchmarking against the microwell format shows that roughly half of the shortfall is attributable to the chromogenic chemistry itself, a limitation that would persist in any format using these reagents, while the remainder reflects the microfluidic platform. The order of reagent interaction with the sample introduces an additional source of variance: introducing the diluted plasma to FXa before the substrate performed significantly better than the reverse order. This is consistent with the prior interaction allowing the anticoagulant to inhibit the enzyme before the substrate becomes available, whereas simultaneous exposure forces substrate and inhibitor to compete immediately for the same active site.

The remaining variance is device-dependent and amenable to engineering optimization. Denaturation during drying can be excluded as a source, since both reagents retained their activity when dried and reconstituted immediately before assay (Supplementary Figure S1 and Table S1). That experiment was performed in microwells rather than on-chip and addressed the drying step alone, so the long-term storage stability of the dried reagents and their robustness within the assembled device were not examined and remain to be established. For the variability observed here, however, the relevant mechanism is the uniformity with which the dried reagents redissolve. Heterogeneous reconstitution of the dried reagents, together with flow inconsistencies arising from partial channel occlusion or fabrication tolerance and variation in the optical path length across reaction chambers, accounts for the remaining error [13,14]. Such inter-device variability remains a primary obstacle to the regulatory approval and clinical translation of microfluidic hemostasis platforms, prompting calls for standardized handling and performance validation protocols within the field [15]. Two established engineering solutions address these issues without altering the underlying assay chemistry. Encapsulation and controlled-release formulations for dried reagents in thermoplastic devices give more uniform reconstitution [16], and transitioning from manual lamination to automated injection molding or additive manufacturing would eliminate the structural misalignments inherent to hand assembly.

Quantitative reporting will require a calibration established in the same medium as the sample. The artificial plasma used for the calibrators does not reproduce the optical background, protein content, and endogenous inhibitor concentration of patient plasma, so an absorbance value cannot be converted to a concentration using an external calibration curve. Two routes are available: calibrators prepared in pooled human plasma or a reference reaction performed within the sample itself, which would generate a calibration point for each measurement without an additional user step. Spike-and-recovery experiments in pooled normal and patient plasma would quantify the effect and are the most direct next step in the analytical validation. The same applies to hemolysis, icterus, and lipemia, each of which absorbs at 405 nm; formal interference testing is required before the assay is applied to unselected clinical samples.

Defining the operating window of this sequence is a natural next step. The order is effective while the sample reaches the second reagent after inhibition is complete, a condition governed by flow time and by the rate at which the dried reagents dissolve. Both were held constant here, and mapping the assay across a range of these values would set the tolerances within which the sequence performs reliably, which is the information a manufacturing specification would need.

### 4.2. Comparison with Existing Point-of-Care Devices

Most published clinical evaluations of POCT devices are based on clot-based tests, and their reported accuracy varies considerably with both device type and anticoagulant (Table 1). Surman et al. compared POCT aPTT with laboratory aPTT in patients receiving heparin infusion and reported poor correlation [17]. The coefficient obtained in the present study (r = −0.58) compares favorably with that report and exceeds several of the clot-based results reported for apixaban and dabigatran. The stronger agreement reported for rivaroxaban and edoxaban on the CoaguChek and Hemochron INR channels indicates the level of performance that a point-of-care anti-Xa device can reasonably aim for and provides a useful target for design improvements. It is difficult to make direct comparisons across these studies due to variations in their analytes, patient populations, and reference methods. Because our assay measures anti-Xa activity directly instead of assessing clotting time, it presents a very different performance profile compared to clot-based devices. The two approaches are therefore likely to be complementary, although further research is needed to determine how well this assay performs for specific DOACs.

DOASENSE provides a urine dipstick for the analysis of DOACs, in which results are indicated by color changes on paper pads. In a recent prospective evaluation in 101 patients with acute ischemic stroke, the dipstick detected clinically relevant anti-Xa activity with high sensitivity, against a median turnaround of 144 min for the corresponding plasma assays [22]. Its readout is nevertheless qualitative and urine-based: interpretation is observer-dependent, abnormal urine color may interfere, and a positive result still requires plasma testing before management is altered [23]. The present device returns a quantitative plasma result in approximately 40 min on the same scale as the laboratory assay, so the two are directly comparable. Harris et al. reported a microfluidic device in which the enzymatic substrate was pre-stored in powder form, although FXa still had to be mixed manually with the sample before loading [24]. Electrochemical approaches are also under development, including an immunosensor that distinguishes FX from FXa for the purpose of DOAC monitoring [25]. To the best of the authors’ knowledge, and on the basis of the comparison with published devices summarized above, the device described here is the first anti-Xa assay chip in which both reagents are pre-stored on-chip; eliminating manual reagent handling is what allows the analytical sequence to be automated within the disposable itself. Because the result is read on a standard microplate reader, the format could be adopted readily by established medical laboratories, and integration of a compact dedicated reader would extend the same assay chemistry to a fully portable platform.

### 4.3. Clinical Role of the Assay

Rapid anti-Xa measurement is of greatest value when the result alters immediate management, as in major bleeding, suspected overdose, assessment before an urgent procedure, or thromboembolism occurring during anticoagulation. It is also of value in critical care, where heparin dosing is adjusted to maintain a target range [26]. In each of these situations, the clinical question is a threshold decision rather than a measurement: whether clinically significant anticoagulant activity is present. This is the question the present device answers effectively.

**Figure 8:**
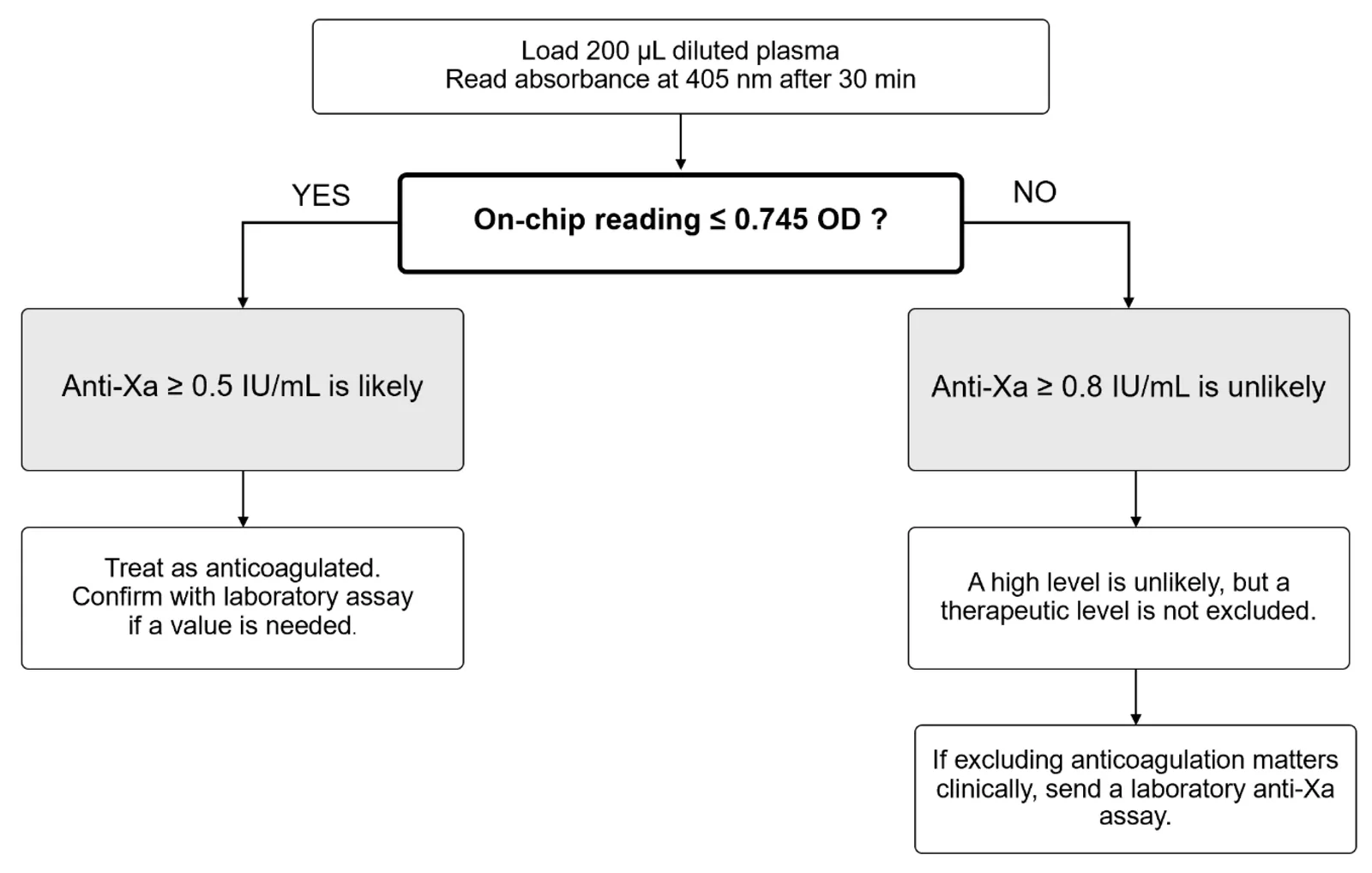
Interpretation of a microfluidic anti-Xa result.

Figure 8 summarizes the practical clinical interpretation of the assay results. An on-chip absorbance reading at or below 0.745 OD indicates that a therapeutic anti-Xa concentration (≥0.5 IU/mL) is likely, a classification that proved correct for 25 of 26 flagged samples. A reading above the cut-off does not support the opposite conclusion. Sensitivity at this threshold was 61%, so a substantial proportion of therapeutically anticoagulated samples were not flagged, and an unflagged result cannot be taken to exclude clinically relevant anticoagulant activity. Even against the higher 0.8 IU/mL threshold, where 28 of 32 unflagged samples were correctly classified, the result indicates only that a high concentration is less likely and does not constitute exclusion. At the present stage of development, both readings are therefore best treated as preliminary, with a standard laboratory anti-Xa assay used for confirmation whenever the result would alter clinical management.

### 4.4. Outlook

Realizing this design as a deployable test requires development on two fronts: analytical and engineering. Three analytical steps would extend the device from classification to measurement. Calibration in matched plasma, or a reference reaction within the sample, would allow concentrations rather than categories to be reported. Interference testing for hemolysis, icterus, and lipemia would define the range of samples to which the assay can be applied, and establishing the shelf life of the dried reagents would determine how the disposable can be stored and distributed.

Two engineering changes would make the device self-contained. Plasma is currently separated by centrifugation; a plasma-separation stage upstream of the first reagent chamber, by membrane filtration or by a microfluidic cell-separation structure, would allow whole blood to be applied directly. Absorbance is read on a laboratory microplate reader with a customized jig; a compact reader built around an LED source and photodiode matched to 405 nm, with the optical path defined by the depth of the reaction chamber, would remove that dependence. The assay operates over an absorbance range of roughly 0.4 to 1.6 OD, which is well within the capability of such a detector. Turnaround can also be shortened. The reaction is substantially complete by 15 min at 25 °C, so the 30 min incubation could be halved (Supplementary Section S1). Incubation at 37 °C accelerates the early part of the reaction, although both temperatures reach 99% of their respective plateaus by 15 min (Supplementary Section S4). Ambient operation has therefore been retained for simplicity, and a heated format may still be worth considering where a shorter time to a stable reading is required.

Three points define the scope of this work. The system as it stands is a microfluidic platform with potential for point-of-care use rather than a finished point-of-care device, and the developments above indicate what would close that gap. The clinical evaluation covered LMWH only; since the assay measures anti-FXa activity, the direct oral anticoagulants are an obvious next target, though performance in those samples remains to be established. The specimens were also de-identified at the source, so patient age, renal function, the LMWH preparation used, and the timing of each sample relative to dosing could not be retrieved. The measured activities span 0.06 to 1.38 IU/mL, indicating that both peak and non-peak samples were included. A cohort with documented clinical characteristics would show how far these results generalize.

## 5. Conclusions

A disposable microfluidic device using pre-stored, dried reagents was developed for the measurement of anti-factor Xa activity, yielding a result in approximately 40 min. Agreement with the automated laboratory assay was moderate on a concentration scale, and the device performed considerably better when used to classify samples: against a reference threshold of 0.5 IU/mL, therapeutically anticoagulated samples were identified with a specificity of 94% and a positive predictive value of 96%. These results establish analytical feasibility and support a preliminary screening role, with clinical utility to be confirmed in an independent cohort. Calibration in matched plasma, integration of plasma separation and a compact optical detector, and evaluation in patients receiving direct oral anticoagulants define the path from the present platform to a deployable point-of-care test.

## Figures and Tables

**Table 1:** Summary of published clinical evaluation reports on anticoagulant point-of-care tests. Statistics are reproduced as reported in each source.

POCT Product	Anticoagulant	Reference Method	Correlation Coefficient	Reference
Hemochron^®^-aPTT	Heparin	Lab aPTT	Poor correlation	[17]
CoaguChek^®^-INR	Edoxaban	UPLC-MS/MS	r = 0.73	[18]
Hemochron^®^-INR	Apixaban	UPLC-MS/MS	r = 0.37	[19]
Hemochron^®^-ACT+			r = 0.48	
Hemochron^®^-ACT-LR			Poor correlation	
Hemochron^®^-aPTT			Poor correlation	
Hemochron^®^-INR	Rivaroxaban	UPLC-MS/MS	r = 0.79	[19]
Hemochron^®^-ACT+			r = 0.78	
Hemochron^®^-ACT-LR			r = 0.399	
Hemochron^®^-aPTT			r = 0.3983	
Hemochron^®^-INR	Edoxaban	UPLC-MS/MS	R^2^ = 0.90	[20]
Hemochron^®^-ACT+			R^2^ = 0.62	
Hemochron^®^-ACT-LR			R^2^ = 0.488	
Hemochron^®^-aPTT			R^2^ = 0.3653	
CoaguChek^®^-PT	Rivaroxaban	Anti-Xa assay	r = 0.82	[21]
	Apixaban		Poor correlation	
	Dabigatran		Poor correlation	

Note: r denotes the Pearson correlation coefficient and R^2^ the coefficient of determination; the two are not directly comparable. The R^2^ values quoted from ref. [20] correspond to r = 0.95, 0.79, 0.70 and 0.60. For context, r = 0.58 obtained with the better-performing protocol in the present study corresponds to R^2^ = 0.34, indicating that approximately one-third of the variance in the reference measurement is accounted for by the on-chip result.

## Data Availability

The individual patient sample results supporting the conclusions of this article are provided as a separate data file and are available from the corresponding author upon request.

## Supplementary Materials

The following supporting information can be downloaded at https://www.mdpi.com/article/10.3390/s26216803/s1, Figure S1: Reaction kinetics after vacuum drying and reconstitution; Table S1: Endpoint absorbance after vacuum drying and reconstitution; Figure S2: Clinical evaluation using the reversed reagent sequence (n = 62), in which the diluted plasma reconstituted the chromogenic substrate before reaching the dried FXa; Table S2: Replicate calibrator measurements; Table S3: Precision of the two assay formats at each calibrator level, derived from the replicate measurements in Table S2; Figure S3: Reaction kinetics at 25 and 37 °C for factor Xa and chromogenic substrate mixed in a microwell plate in the absence of heparin; Table S4: Reaction kinetics at 25 and 37 °C, expressed as absorbance and as a percentage of the plateau for each temperature.
