# Supplementary material for: Single-Step Microfluidic Anti-Factor Xa Assay: Development and Clinical Evaluation in Patients on Low-Molecular-Weight Heparin
Source: Sensors (Basel). 2026 Oct 28;26(21):6803. doi: 10.3390/s26216803 (PMC13765726; doi:10.3390/s26216803)
Supplement: Supplementary file 1 [file sensors-26-06803-s001.zip › sensors-4552983-supplementary.pdf]

Supplementary Materials

Single-Step Microfluidic Anti-Factor Xa Assay: Development and Clinical Evaluation in Patients on Low-Molecular-Weight Heparin

S1. Retention of reagent activity after vacuum drying and reconstitution

The two assay reagents are supplied as ready-to-use liquid formulations (STA-Liquid Anti-Xa, Stago). The package insert specifies the active components, but does not disclose the complete formulation, so the presence of proprietary stabilizers can be neither confirmed nor excluded. No lyoprotectant, carbohydrate stabilizer or carrier protein was added before drying.

To determine whether the drying step itself compromises activity, each reagent was dried and reconstituted separately and assayed against its fresh counterpart. Chromogenic substrate (50 µL) and bovine factor Xa (50 µL) were dispensed into separate microwells and dried under vacuum until solid, then reconstituted in 50 µL of deionized water. Three conditions were compared, each in a total volume of 110 µL: reconstituted substrate with fresh FXa; reconstituted FXa with fresh substrate; and both reagents fresh, as the control. No heparin was present, so the signal reflects the maximum attainable enzyme activity. Absorbance at 405 nm was recorded at 1, 3, 5, 7, 10, 15, 20, 30 and 40 min at room temperature.

All three conditions followed the same reaction course and converged on a common endpoint (Figure S1, Table S1). Relative to the fresh control, the plateau absorbance was 99.1% when the substrate had been dried and 103.6% when the FXa had been dried, both within the run-to-run variation of the microwell assay. Had drying denatured either reagent, a proportionally lower plateau would be expected; none was observed. Reconstituted FXa reached 95% of its plateau at 15 min compared with 10 min for the fresh reagents, indicating a modest delay in dissolution rather than a loss of activity.

The kinetic curves also show that the reaction is substantially complete well before the 30 min incubation used in the main protocol: all three conditions reached 95% of their respective plateaus within 10 to 15 min. The 30 min interval was retained for consistency with the manual microwell and automated analyzer protocols rather than because it is required by the on-chip reaction, and these data indicate that it could be shortened without loss of signal.

These results indicate that the vacuum-drying protocol does not compromise the activity of either reagent. The between-chip variability reported in the main text is therefore attributed to non-uniform dissolution and to fabrication tolerance rather than to enzyme denaturation. The experiment was performed in microwells rather than on-chip, and so isolates the effect of drying and reconstitution from the geometry of the device.

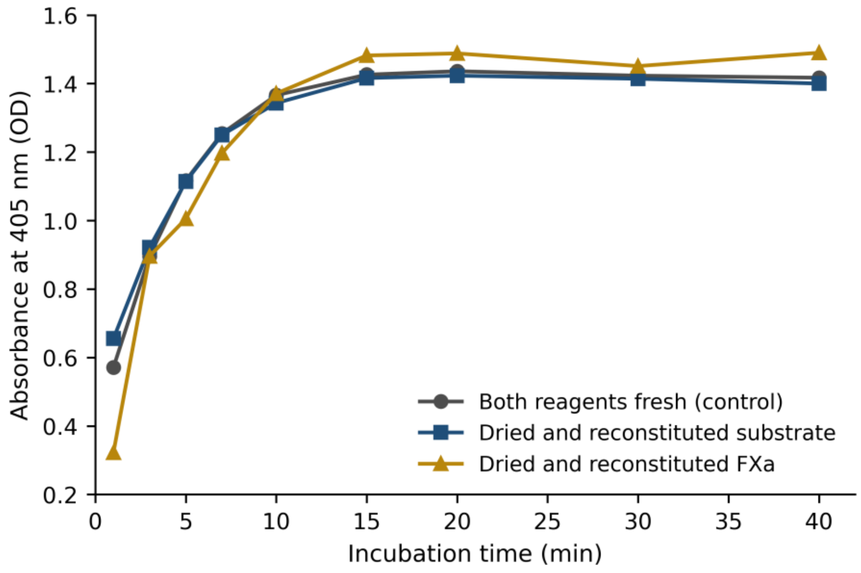

Figure S1. Reaction kinetics after vacuum drying and reconstitution. Absorbance at 405 nm in the absence of heparin, for chromogenic substrate dried and reconstituted before use, factor Xa dried and reconstituted before use, and both reagents used fresh. Each point is a single measurement.

Table S1. Endpoint absorbance after vacuum drying and reconstitution. The plateau is the mean of the 20, 30 and 40 min readings.

Condition	Plateau absorbance (OD)	Relative to control (%)	Time to 95% of plateau (min)
Both reagents fresh (control)	1.425	100.0	10
Dried and reconstituted substrate	1.412	99.1	10
Dried and reconstituted FXa	1.476	103.6	15

S2. Clinical evaluation using the reversed reagent sequence

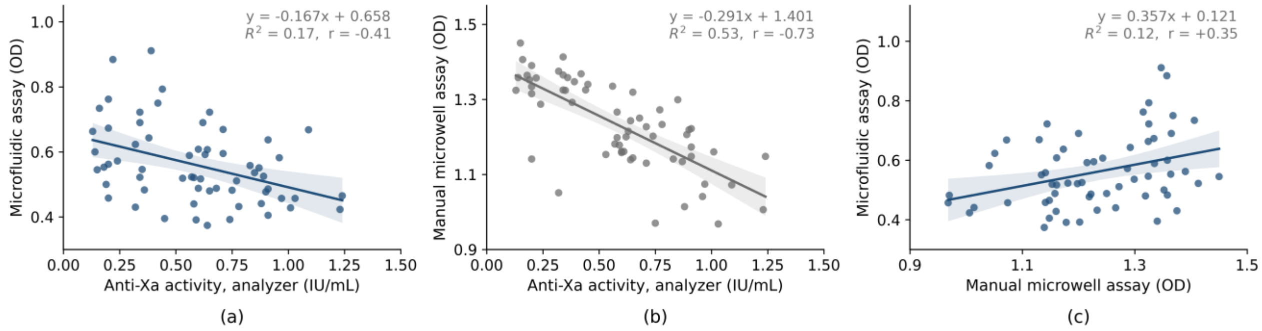

Figure S2. Clinical evaluation using the reversed reagent sequence (n = 62), in which the diluted plasma reconstituted the chromogenic substrate before reaching the dried FXa. (a) Correlation between microfluidic assay OD and anti-Xa activity measured on the automated analyzer; (b) correlation between manual microwell assay OD and the same analyzer result; (c) correlation between microfluidic and manual microwell assay OD. Shaded bands are 95% confidence intervals of the fitted regression line.

S3. Calibrator and precision data

Table S2. Replicate calibrator measurements. Each value is an independent run, performed on a separate chip for the microfluidic assay and in a separate well for the manual assay. The two standard curves were generated in separate experiments.

Calibrator (IU/mL)	Microfluidic assay (OD)	Manual microwell assay (OD)
0.00	0.906	1.481
	1.310	1.509
	1.084	1.503
	0.940	1.451
	0.971	1.419
	0.942	1.350
	0.903	1.335
	1.064	1.294
	0.942	1.268
	—	1.298
0.41	0.838	1.358
	0.909	1.458
	0.951	1.370
	0.930	1.209
	1.234	1.244
	0.909	1.431
	0.765	1.241
	1.134	1.209
	—	1.290
	—	1.245
0.63	0.902	1.275
	1.007	1.288
	0.810	1.300
	1.024	1.292
	0.722	1.363
	0.831	1.349
	0.890	—
	0.837	—
	0.837	—
1.76	0.703	0.997
	0.635	0.976
	0.791	0.889
	0.624	0.997
	0.636	1.052
	0.673	1.040
	0.674	—

Table S3. Precision of the two assay formats at each calibrator level, derived from the replicate measurements in Table S2. CV denotes the coefficient of variation.

Calibrator (IU/mL)	Microfluidic mean ± SD (n)	CV (%)	Microwell mean ± SD (n)	CV (%)
0.00	1.007 ± 0.130 (9)	13.0	1.391 ± 0.092 (10)	6.6
0.41	0.959 ± 0.153 (8)	16.0	1.305 ± 0.092 (10)	7.1
0.63	0.873 ± 0.096 (9)	10.9	1.311 ± 0.036 (6)	2.7
1.76	0.677 ± 0.058 (7)	8.5	0.992 ± 0.058 (6)	5.8
**Pooled SD (OD)**	**0.115**	—	**0.074**	—

S4. Effect of incubation temperature on reaction kinetics

The assay is performed at ambient laboratory temperature so that no incubator or heating element is required. To establish whether incubation at body temperature would shorten the reaction usefully, the time course was compared at 25 and 37 °C. Factor Xa (50 µL) and chromogenic substrate (50 µL) were mixed in a microwell plate in the absence of heparin and incubated at the stated temperature, and absorbance at 405 nm was recorded at 1, 3, 5, 7, 10, 15, 20, 30 and 40 min.

The reaction proceeded slightly faster at 37 °C over the first 10 min, reaching 92% of its plateau at 7 min compared with 85% at 25 °C (Figure S3, Table S4). The advantage did not persist. Both temperatures reached 95% of their respective plateaus at 10 min and 99% at 15 min, and the endpoint absorbance differed by 0.024 OD, or 1.9%. Raising the temperature therefore brings forward the point at which the reaction is substantially complete by a few minutes at most, and does not alter the incubation time that the assay requires.

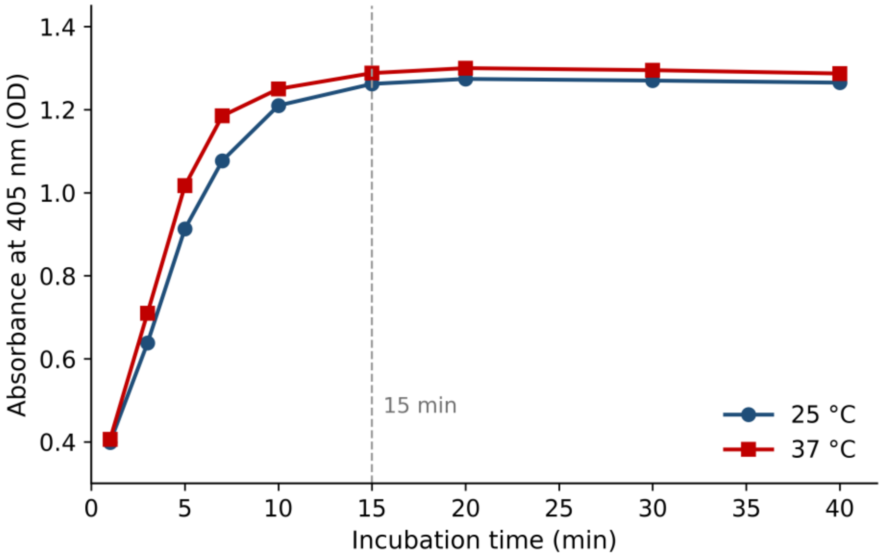

Figure S3. Reaction kinetics at 25 and 37 °C. Absorbance at 405 nm for factor Xa and chromogenic substrate mixed in a microwell plate in the absence of heparin. The dashed line marks 15 min, by which both conditions had reached 99% of their respective plateaus. Each point is a single measurement.

Table S4. Reaction kinetics at 25 and 37 °C, expressed as absorbance and as a percentage of the plateau for each temperature. The plateau is the mean of the 20, 30 and 40 min readings.

Time (min)	25 °C (OD)	% of plateau	37 °C (OD)	% of plateau
1	0.399	31%	0.406	31%
3	0.638	50%	0.710	55%
5	0.913	72%	1.017	79%
7	1.077	85%	1.185	92%
10	1.210	95%	1.250	97%
15	1.262	99%	1.288	100%
20	1.274	100%	1.300	100%
30	1.270	100%	1.295	100%
40	1.265	100%	1.287	99%
**Plateau**	**1.270**	**100%**	**1.294**	**100%**

Individual patient sample results are provided as a separate data file, available from the corresponding author on request.
